# Multidisciplinary users oriented approach in the community mental health care

**DOI:** 10.1192/j.eurpsy.2024.1239

**Published:** 2024-08-27

**Authors:** G. Racetovic, S. Grujic Timarac, M. Latinovic

**Affiliations:** ^1^ Community Mental Health Center, HEALTH CENTER Prijedor; ^2^Psychiatric Department, Hospital “Dr. Mladen Stojanovic”, Prijedor; ^3^Deputy of the Minister, Ministry of Health of Republic of Srpska, Banja Luka, Bosnia and Herzegovina

## Abstract

**Introduction:**

For more than 25 years, Bosnia and Herzegovina (BH) has maintained and improved reform processes in the field of mental health (MH) care. In the last 13 years, the results of the reform are visible, and they focused on the quality of services oriented to people with mental disorders (PMD), as well as raising the quality of specific services to the general population. The developed network of community mental health centers (CMHC) as the leading concept of the reform (community psychiatry) enabled affordable, timely and adequate protection of mental health in whole country, while relying on other resources, primarily clinical and hospital capacities and centers for social work.

**Objectives:**

Reform orientation had several directions: broad promotion and prevention, protection of the rights of the people with mental health disorders, quality education and more specific qualifications and competencies of mental health professionals and public promotion as a part of destigmatization.

**Methods:**

Overview of the mental health reform in last two decades.

**Results:**

In last more than 25 years reforming processes were focused mainly to users of mental health services. Specific legislative was created and recently upgraded trough Mental Health Protection Law, as unique document focused to users, mental health professionals and services, and general population as well. Users organization were formed and have important role in creation of further directions in the reform. Main principles in daily care for users are community based with case management and intersectoral collaboration trough joint discharge planning. Also, special focus is given to media reporting about mental health as well as to children and adolescents, prenatal psychiatry and elderly.

**Conclusions:**

Republic of Srpska and Bosnia and Herzegovina made significant steps and visible changes in the quaity of mental health services focused to users needs. Further activities will be oriented to continuous destigmatization of the people with mental health disorders, psychiatry and mental health professionals and further implementation of results achieved in reforming processes in our county.

**Disclosure of Interest:**

None Declared

